# Determination of the Round Window Niche Anatomy Using Cone Beam Computed Tomography Imaging as Preparatory Work for Individualized Drug-Releasing Implants

**DOI:** 10.3390/jimaging7050079

**Published:** 2021-04-26

**Authors:** Farnaz Matin, Ziwen Gao, Felix Repp, Samuel John, Thomas Lenarz, Verena Scheper

**Affiliations:** 1 Lower Saxony Center for Biomedical Engineering, Implant Research and Development (NIFE), Department of Otorhinolaryngology, Head and Neck Surgery, Hanover Medical School, Stadtfelddamm 34, 30625 Hannover, Germany; Gao.ziwen@mh-hannover.de (Z.G.); lenarz.thomas@mh-hannover.de (T.L.); scheper.verena@mh-hannover.de (V.S.); 2 Cluster of Excellence “Hearing4all” EXC 1077/1, 30625 Hanover, Germany; 3 OtoJig GmbH, 30625 Hanover, Germany; repp.felix@otojig.com (F.R.); john.samuel@hoersys.de (S.J.); 4 HörSys GmbH, 30625 Hanover, Germany

**Keywords:** cone beam computed tomography, round window niche, round window membrane, individualized implant

## Abstract

Modern therapy of inner ear disorders is increasingly shifting to local drug delivery using a growing number of pharmaceuticals. Access to the inner ear is usually made via the round window membrane (RWM), located in the bony round window niche (RWN). We hypothesize that the individual shape and size of the RWN have to be taken into account for safe reliable and controlled drug delivery. Therefore, we investigated the anatomy and its variations. Cone beam computed tomography (CBCT) images of 50 patients were analyzed. Based on the reconstructed 3D volumes, individual anatomies of the RWN, RWM, and bony overhang were determined by segmentation using 3D Slicer^TM^ with a custom build plug-in. A large individual anatomical variability of the RWN with a mean volume of 4.54 mm^3^ (min 2.28 mm^3^, max 6.64 mm^3^) was measured. The area of the RWM ranged from 1.30 to 4.39 mm^2^ (mean: 2.93 mm^2^). The bony overhang had a mean length of 0.56 mm (min 0.04 mm, max 1.24 mm) and the shape was individually very different. Our data suggest that there is a potential for individually designed and additively manufactured RWN implants due to large differences in the volume and shape of the RWN.

## 1. Introduction

**Figure 1:**
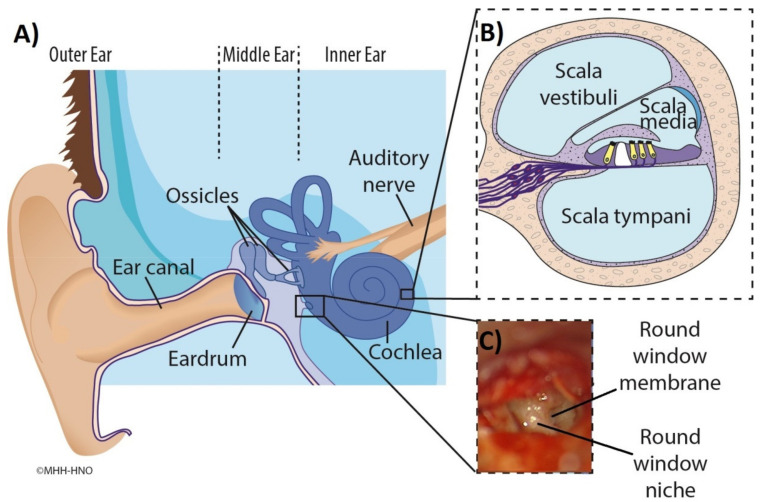
Illustration of healthy ear anatomy. Main figure (**A**): Structure of the middle ear containing the tympanic membrane (eardrum), round window niche, and auditory ossicles. The cochlea and auditory nerve are also shown. Inset (**B**): (top right) Cross-section of the cochlea illustrating the three fluid filled compartments scala vestibuli, scala media with sensory cells (yellow), and scala tympani. Inset (**C**): (bottom right) Intraoperative microscopic appearance of the round window region seen through facial recess with focus on the round window niche and round window membrane.

Over the last decades, there has been increasing interest in the possibility of treating inner ear disorders, such as idiopathic sudden sensorineural hearing loss (ISSHL) and Ménière’s disease (MD), by application of medication into the middle ear. Substances can pass from the middle to the inner ear via diffusion through the semipermeable round window membrane (RWM), which, in the human, is located deep in a recess, the round window niche (RWN), between the middle ear and inner ear (Figure 1) [1,2]. Various pharmacological treatments and drug delivery strategies have been proposed. Drugs can be administrated directly into the middle ear cavity, i.e., intratympanically (IT), by needle injection through the tympanic membrane, or injection through a ventilation tube placed into the tympanic membrane. Intratympanically applied drugs are absorbed into the inner ear perilymph through the RWM and, to a small amount, through the oval window. Comparing the drug concentrations in the perilymph after systemic administration and IT injection, the latter results in 425- to 1270-fold higher drug levels, corrected for dose [3,4] by bypassing the blood-labyrinthine barrier and first pass metabolism.

Biodegradable polymers have also been suggested as drug delivery strategies for the treatment of inner ear disorders [5]. To sustain a prolonged drug delivery to the inner ear and achieve a limited degradation of the drug, biomaterials, such as hydrogels, have been applied. Soluble drugs can be loaded into hydrogels and placed at the RWM. Once applied, the hydrogel releases the drug by hydrolysis of the matrix or by diffusion out of the matrix [6].

Another approach of drug delivery to the inner ear is the use of a microcatheter. The distal catheter end rests inside the patient’s round window niche (RWN) and the applied drug has contact with the RWM without damaging it [7,8,9]. The effective rate of the microcatheter drug perfusion in patients with ISSHL who failed a conventional treatment ranged from 38 to 53 percent in prospective studies. However, there are no placebo controlled trials [10].

Local drug delivery has been used more frequently over the last years compared to systemic treatment in order to avoid negative side effects and achieve higher concentrations of the agent in the inner ear. However, there are some disadvantages of the before mentioned strategies, mainly that large portions of the administered drug do not come into contact with the RWM. It is instead absorbed by the mucosa of the middle ear or evacuated from the middle ear space by the Eustachian tube. In either case, the drug is unavailable to diffuse into the inner ear [11,12]. Additionally, pseudomembranes covering the opening of these access routes leading to a not well determined contact duration of the drug to the RWM.

Drug delivery would be sustained if the drug remained on the RWM which is, in part, already achieved by gel-matrix based RWM delivery. However the matrix based delivery is not yet controlled in terms of matrix size and drug load [6,13,14].

One new approach that may provide the potential for an efficient and safe administration route of controlled drug delivery via the RWM is by developing implants with optimal drug loading for individual needs, fitting exactly in the RWN for optimal pharmacokinetics and safety reasons. Those implants have to be manufactured in the individual shape of the corresponding RWN for accurate placement directly on the RWM. Only by this means will the implants optimize the attachment of the drug releasing matrix surface on the RWM for sustained release. In contrast to gel matrices the implant should keep the drug in place for a few weeks to achieve a prolonged dwell time and keep the drug delivery duration extended. Therefore the aim of the present work was to radiologically assess the morphological variations of the RWM and RWN to determine the necessity of individualized implants, which may improve the local drug delivery to the inner ear. Another important aim of this study was to verify whether the shape of the RWN could be segmented using cone beam computed tomography (CBCT) datasets of unilateral temporal bones of clinical quality as a basis for the development of software analysis tools for these novel implants.

## 2. Material and Methods

To determine the anatomical variability of the RWN, 50 anonymized CBCT datasets of unilateral temporal bones of patients were included in the study. The study was conducted in accordance with the Declaration of Helsinki. The protocol for the use of the patient’s data for this retrospective study was approved by the Ethics Committee of Hanover Medical School (Project identification code 3699-2017). Patients were retrospectively selected based on having no history of oto-surgical manipulation before imaging. Due to the retrospective design, no written information was given to the patients of the study group. However, only patients who agreed to the general use of their data were selected. All patient data were anonymized and de-identified prior to the retrospective analysis. The scans were conducted within the clinical routine at the Hanover Medical School independent of this study. The selection process of the patients to be included in the study was random in general, but malformed or diseased (ossified) cochleae were excluded. The patients were scanned using a 3D ACCUITOMO 170 Digital CBCT scanner (J. Morita Tokyo Mfg. Corp., Tokyo, Japan). Resulting CBCT images were reconstructed and exported as Digital Imaging and Communications in Medicine (DICOM) Data using the i-Dixel software (J. Morita Tokyo Mfg. Corp., Tokyo, Japan) with a voxel size of 0.08 × 0.08 × 0.08 mm [15].

The reconstruction and segmentation of the DICOM-data from CBCT was performed using 3D Slicer 4.11 version 4.11 (http://www.slicer.org) nightly [16].

**Figure 2:**
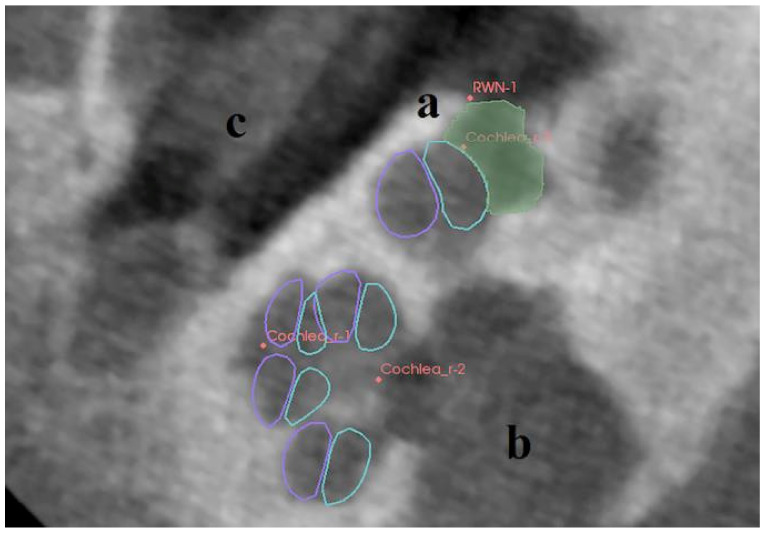
Using 3D Slicer and a custom made plug-in, points were placed manually at the midmodiolar apex (Cochlea_r-1), at midmodiolar basal turn (Cochlea_r-2), at the RWM (Cochlea_r-3) and on the tip of the bony overhang (RWN-1). These points allowed the determination of the midmodiolar axis by fitting a mean model of the ST (in turquoise) and the SV (in purple) in the CBCT data. The manually segmented RWN is shown in green. (**a**) bony overhang, (**b**) inner ear canal with N. vestibulocochlearis and N. facialis, (**c**) middle ear mucosa.

**Figure 3:**
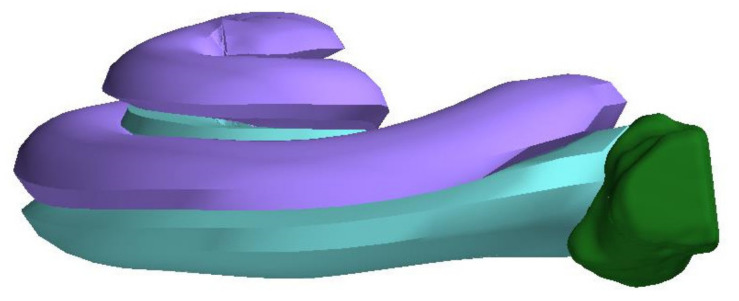
Three-dimensional reconstruction of the segmented cochlear with the ST (turquoise) and the SV (purple) and the RWN (green) in 3D Slicer.

A threshold supported manual paint segmentation technique was chosen for segmentation of the datasets using 3D Slicer. A custom build plug-in for 3D Slicer was used to determine the midmodiolar axis by fitting a model of the scala tympani (ST) and the scala vestibuli (SV) (Figure 1B) in the DICOM data. This model includes the mean cross-sections of the scales based on *n* = 47 samples [17] and additionally helped to determine the boundary of the RWN to the ST. For a determination of location, orientation and scalings, three points were placed at the cochlea: One was placed at the midmodiolar apex, the other one at the midmodiolar basal turn of the cochlea, and the third one at any point of the RWM (Figure 2). Moving these points leads to an update of the visualized model, thus allowing matching the ST/SV model to the bone cochlear interface. These three points define a common coordinate system relative to the cochlea so that magnitudes like the depth of the RWN and the length of the bony overhang could be determined in comparable directions [18]. The *z*-axis is defined parallel to the midmodiolar axis. The *x*-axis points from the midmodiolar axis to the round window, and a *y*-axis orthogonal to *x*-axis and *z*-axis. For defining the dimensions of the RWN, it was manually segmented in each slicing plane of the datasets. To get a comparable measurement of the RWN regarding its protrusion into the middle ear, an additional point (RWN_1) was set on the bony tip of the bony overhang (Figure 2). The distance from the point at the tip of the bony overhang to the midmodiolar axis is used to define the border of the RWN to the tympanic cavity: Using this distance to define a cylinder around the midmodiular axis, the manual segmentation is cut such that it does not extend beyond this cylinder. As a result of the described process, we were able to provide three-dimensionally reconstructed sets of the segmented cochleae and RWN of the human temporal bones, as exemplarily shown in Figure 3.

**Figure 4:**
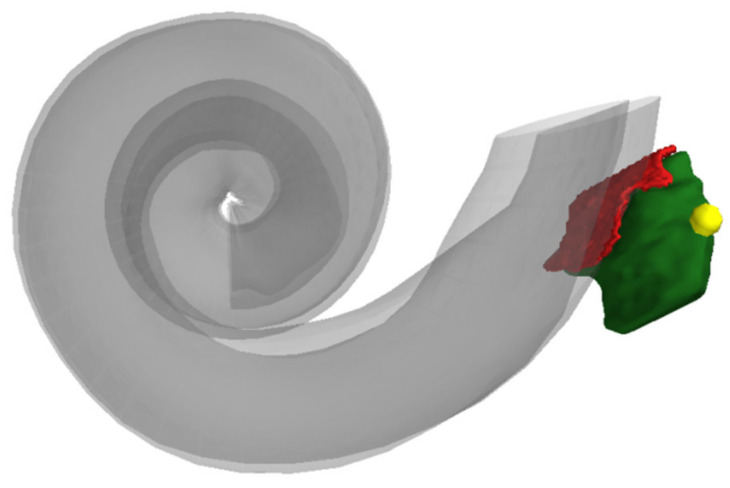
Three-dimensional reconstruction of the segmented RWN (in green) and the RWM (in red). The yellow point marks the point set on the tip of the bony overhang.

Subsequently, the segmentation data were transformed into voxel data. With this, we determined the interface of the RWN to the ST to measure the area of the RWM. The 3D image surrounding the RWN segmentation was automatically set to a threshold to identify the surface of the bone that is in contact with the RWN, allowing separating the surface of the RWM (Figure 4).

The anatomy of the RWN and RWM was studied with respect to the volume of the RWN as well as the surface of the RWM. Additionally, several extends in directions defined within the earlier introduced common coordinate system were measured: The depth *d* of the RWN as extend along the z- axis. The bony overhang *oh* was defined as Y position of the voxel of the RWN furthest to the point at the tip of the bony overhang.

All these quantities were analyzed using Prism 5.02 (GraphPad Software, Inc., La Jolla, CA, USA). D’Agostino and Pearson omnibus normality test was used to evaluate if a Gaussian distribution exists. Volume and RWM area data were grouped for sex and compared using unpaired t-test. Pearson correlation test was applied to analyze volume and RWM area dependencies to age. The segmented left side RWNs (16 of the 50 temporal bones included in the presented study) were mirrored to the right to allow for direct comparison of all analyzed shapes.

## 3. Results

**Figure 5:**
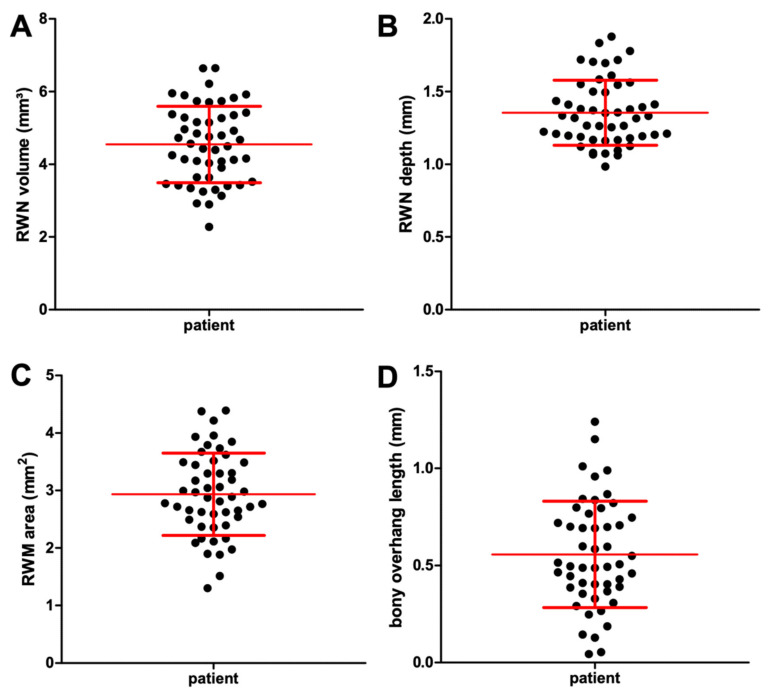
Graphical illustrations showing the wide range of the individual anatomical dimensions of the round window niche (RWN) volume (**A**) and depth (**B**), round window membrane (RWM) area (**C**) and bony overhang length (**D**) derived from segmentations of *n* = 50 clinical CBCT datasets. The individual data points, as well as the mean and standard deviation (red lines), are plotted.

**Figure 6:**
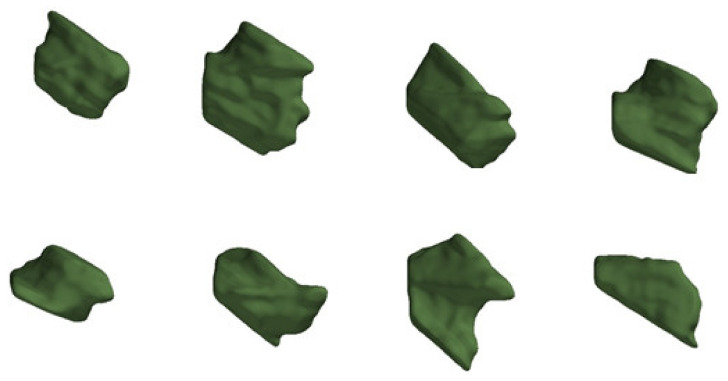
Each green shaped body shows a RWN of an individual patient. The upper part of each body shows the area of the RWM. This exemplary illustration of eight different RWN demonstrates the large observed variability of the RWN shape.

**Figure 7:**
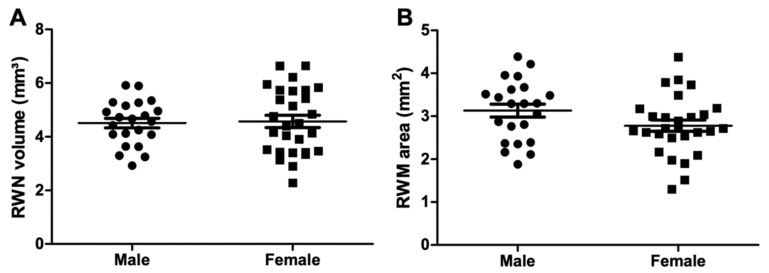
The RWN volume (**A**) and RWM area (**B**) did not differ between male and female patients.

The CBCT imaging of temporal bones of 50 patients (22 male, 28 female) with a mean age of 53 years (min 13 years old, max 92 years old) were evaluated. RWN volume and depth, RWM area and bony overhang length were normally distributed. The RWN volume and area were grouped in regard to age and sex and tested for Gaussian distribution, which was still proven. A large individual anatomical variability of the RWN with a mean volume of 4.54 mm^3^ (min 2.28 mm^3^, max 6.64 mm^3^) was measured (Figure 5A). The depth of the RWN from the RWM to the bony overhang ranged between 0.98 and 1.88 mm (mean: 1.35 mm) (Figure 5B). The area of the RWM ranged from 1.30 to 4.39 mm^2^ (mean: 2.93 mm^2^) (Figure 5C). The bony overhang had a mean length of 0.56 mm (min 0.04 mm, max 1.24 mm) (Figure 5D). The form of the niche varied widely between the patients (Figure 6). Table 1 reports the mean, SD, minimum and maximum values for each measured parameter of the 50 segmented CBCT datasets. Grouping the RWN volume data for sex resulted in mean volume values of 4.50 ± 0.82 mm^3^ in male and 4.57 ± 1.21 mm^3^ in female, respectively. Grouping the RWM area data for sex resulted in mean area values of 3.13 ± 0.71 mm^2^ in male and 2.77 ± 0.69 mm^2^ in female, respectively. No sex-specific differences in volume or area were observed (volume: *p* = 0.83; area: *p* = 0.08; Figure 7).

No statistically significant correlation of the RWM area (Pearson r = −0.22; R^2^ = 0.05; *p* = 0.11) and the RWN volume (Pearson r = −0.16; R^2^ = 0.02; *p* = 0.26) to age were observed.

## 4. Discussion

Idiopathic sudden sensorineural hearing loss and MD are mainly treated using pharmacological therapies with a special interest in local delivery of drugs to the inner ear. Local drug delivery to the inner ear is very challenging but superior to systemic treatment due to the reduction of side effects and higher drug concentration in the perilymph. However, applying a drug into the middle ear does not guarantee reaching to the RWM or subsequently to the cochlear. Many anatomic obstructions, such as adhesions, postoperatively aroused scar tissue, overhanging RWN, the false membrane, or thickened membranes, can block the RWM [19,20]. A study of 202 temporal bones reported that 11% of RWNs contained fibrous tissue or fat and another 21% showed a false membrane [1]. Silverstein found out in an examination of 41 RWNs via tympanostomy and middle ear endoscopy that five were completely obstructed and seven were partially obstructed [21]. However, even after overcoming anatomic obstructions in the middle ear, problems with round window permeability for compounds, the loss of medications into the nasopharynx via the Eustachian tube, and drug pharmacokinetics within inner ear fluids can still vary significantly [12,22].

The anatomy of the RWN/RWM region and their surrounding structures respectively anatomical boundaries [23,24] have previously been studied using either dissected cadaveric specimens or histological sections or radiologic imaging of temporal bones and demonstrated significant inter-individual variability [25,26,27,28,29,30]. A recent study conducted by Canzi et al. (2019) aimed at radiologically assessing the morphological variations of the RWN, and relating the imaging findings to their endoscopic anatomy. Three anatomic variations were predominantly described based on the coronal CT scan reconstructions: “Cylindrical-type” (51.7%), “j-type” (32.3%), and “truncated cone- type” (16%) [31]. With the findings of our study, we are adding new information to the relevant literature regarding the anatomical complexity of the RWN, because this study identifies a large sample of three-dimensional reconstructions and measurements the variations in volume and shape of the RWN. Mean volume was found to be 4.54 mm^3^, with large variations in volumes ranging from 2.28 to 6.64 mm^3^. However, it has to be stressed that the nature of the RWN is open towards the middle ear and therefore, the border is not clearly defined. We mitigated this by placing two things: First, we used a multi-planar reconstruction (MPR) aligned to the cochlear coordinate system [18] and second, we located a point on the bony overhang to be used to construct a more defined cut-off of the niche. Despite this, we expect a certain variability, that we did not further investigate, based alone on the manual placement of this point. We found no statistically significant differences in the RWN/RWM according to sex, corroborating the results of previously published studies [28,31,32]. However, the variation in the anatomy of the human RWN may influence the approach of local drug delivery and designs of implants aimed at targeting this region.

Over the last years, personalized medicine has emerged, tailoring specific treatment modalities to individual patient attributes [33]. This should be applied to pharmaceuticals and medical devices for the treatment of inner ear diseases via the RWN approach. Understanding the penetration of drugs through the RWM into the ST is of pharmaceutical importance. Our data set reports a high variability of the RWM area. With some individuals having a very little area of 1.30 mm^2^ and others with a three times larger area of about 4.3 mm^2^, it is obvious that the diffusion process, the drug concentration in perilymph, and finally, the biological effects in the inner ear, will vary between patients when the same drug amount is applied to the membrane. The reported three times larger RWN volume in some patients (6.64 mm^3^) compared to others with a smaller volume of about 2.28 mm^3^ suggests that the drug amount given to the one patient highly differs from the amount applied to the other patient when the RWN is filled with a matrix without controlling the matrix volume and drug load. Knowledge of the individual RWM area and RWN volume will allow the design of personalized implants with optimal drug load for the individual needs. Additionally, the individual RWN shape and the variability of the bony overhang reported in this study involve the risk of traumatizing sensitive structures like the RWM. We could not group the segmented shapes of the RWN into a few classes based on similarity, which would be a prerequisite for ready-made size-tailored implants but found wide variability in size and shape, which requires individually designed and built implants and excludes the use of one size fits all implants. We suggest that novel individualized drug-releasing implants should be developed. Additive manufacturing of drug-loaded individualized implants seems like a promising approach that has not been applied to the RWN/RWM region yet. Additive manufacturing, also referred to as 3D printing, enables to create implants adjusted to the individual anatomical needs of a patient [34,35]. Furthermore, 3D printed drug delivery devices that fit in the RWN may lead to more reliable results in future studies on local drug delivery to the inner ear and therefore, to a benefit for MD and ISSHL patients. Next to atraumatic implantation, by avoiding implantation of too large implants, those implants may give a defined contact area and therefore, a controlled and individualized local drug application (dose-release-curve). And finally, individualized implants may reduce the risk of dislocation of the RWN implant.

Today, it is unknown if a larger RWM area or RWN volume are more likely to result in more effective treatment or if, in those cases, the relatively higher drug concentration in the inner ear will be too high, leading to toxic effects rather than therapeutic benefits. Since up to now it is unclear what exactly are the clinically relevant drug concentrations needed to be locally applied, it is not possible to state how much of which drug has to be loaded in which implant volume. However, the possibility of adjusting the shape, volume, and drug load of RWN implants based on the here presented data, e.g., by additive manufacturing will allow precise local drug delivery. Future pharmacokinetic studies using those precise implants will allow a more controlled local drug delivery, hopefully resulting in outcomes of reduced variability compared to today’s studies. Those future investigations will show if implants, individually manufactured for a specific RWN, will lead to beneficial clinical outcomes in patients. Additionally, they should elucidate if individualized implants are needed for all patients or maybe only for the most difficult cases that correspond to certain values.

## 5. Conclusions

Using our 3D Slicer plug-in for segmentation of the cochlea, our data demonstrate anatomical variability of the RWN volume, bony overhang, and RWM area. For optimal local treatment of inner ear diseases, such as ISSHL and MD, this anatomical individualism should be taken into account. Additive manufacturing may be applied to build individualized drug-eluting RWN implants and future studies need to show if this will improve the outcome of local inner ear therapy.

## Figures and Tables

**Table 1:** Overview of the mean, minimum (min) and maximum (max) results from the *n* = 50 clinical CBCT datasets. Vol = Volume RWN in mm^3^, A = area RWM in mm^2^, *oh* = bony overhang in mm, *d* = depth RWN in mm, std: standard deviation.

	Mean	Min	Max	Std
Vol	4.54	2.28	6.64	1.05
A	2.93	1.30	4.39	0.72
*oh*	0.56	0.04	1.24	0.27
*d*	1.35	0.98	1.88	0.22

## Data Availability

The data is presented within the article.
